# Dissociative Hypomanic Switch: The Importance of Longitudinal Follow-Up

**DOI:** 10.1192/j.eurpsy.2026.11106

**Published:** 2026-07-31

**Authors:** J. G. Ruiz, C. P. C. Alonso, M. M. Muñoz, E. L. Bardón

**Affiliations:** Psiquiatría, Caule, León, Spain

## Abstract

**Introduction:**

Dissociative Identity Disorder (DID) is characterized by the presence of two or more distinct identities or personality states, accompanied by amnesia and behavioral disturbances. Bipolar disorder, on the other hand, is defined by the alternation between episodes of mania or hypomania and major depression, with variability in the duration and intensity of episodes. However, an increasing number of cases of rapid-cycling bipolar disorder have recently been described, whose symptoms may overlap with or be mistaken for those of DID.

**Objectives:**

The aim of this report is to describe the clinical course of a patient initially presenting with dissociative symptoms, later re-evaluated as a case of rapid-cycling Bipolar II Disorder, and to highlight the diagnostic challenges between dissociative and affective disorders.

**Methods:**

We present the case of a 67-year-old woman living with her husband and son. Her psychiatric history began at the age of 30 with an anxious-depressive episode. She had two previous short hospital admissions (2 days each) due to dissociative episodes. During a third admission, she presented with another dissociative episode showing clinical features that overlapped with a hypomanic episode.

On mental status examination at admission, the patient was conscious, oriented, partially approachable, and partially cooperative. Psychomotor restlessness was noted, along with mild suspiciousness and hypervigilance. Speech was accelerated, pressured but interruptible, and she became vociferous when confronted with her symptoms. She displayed a slightly hyperthymic mood with inappropriate/unmotivated laughter. There were no abnormalities in the course or content of thought, nor in perception. No suicidal ideation was present.

**Results:**

Given the coexistence of dissociative symptoms and mood instability, the differential diagnosis was reconsidered. The possibility of rapid-cycling Bipolar II Disorder with hypomanic episodes was proposed. Following the introduction of a mood stabilizer in addition to her usual treatment, the patient has remained clinically stable, with no further dissociative or hypomanic episodes reported to date.

**Conclusions:**

This case illustrates the diagnostic complexity in differentiating dissociative presentations from affective disorders, particularly in the context of rapid-cycling Bipolar II Disorder. Careful longitudinal assessment and therapeutic adjustment are crucial to achieve clinical stability and prevent misdiagnosis.

**Disclosure of Interest:**

None Declared

